# Pro-inflammatory RNA:DNA Hybrids Are p53 Independently Boosted by Hyperbaric Oxygen: a Subcellular Distribution Analysis by Automated Quantitative Imaging

**DOI:** 10.1007/s11307-022-01778-2

**Published:** 2022-10-19

**Authors:** Ilaria De Santis, Michele Zanoni, Sara Pignatta, Pasquale Longobardi, Anna Tesei, Alessandro Bevilacqua

**Affiliations:** 1 Interdepartmental Centre Alma Mater Research Institute on Global Challenges and Climate Change (Alma Climate), University of Bologna, I-40126 Bologna, Italy; 2 Biosciences Laboratory, IRCCS Istituto Romagnolo per lo Studio dei Tumori (IRST) “Dino Amadori”, I-47014 Meldola, Italy; 3 Hyperbaric Centre, I-48124 Ravenna, Italy; 4 Advanced Research Center on Electronic Systems (ARCES) for Information and Communication Technologies “E. De Castro”, University of Bologna, I-40125 Bologna, Italy; 5 Department of Computer Science and Engineering (DISI), University of Bologna, I-40136 Bologna, Italy

**Keywords:** Cancer, Oxidative stress, Oxygen therapy, Radiotherapy, Tumour microenvironment, Inflammation, Automated quantitative imaging

## Abstract

**Purpose:**

RNA:DNA hybrids are co-transcriptional products with acknowledged cytoplasmic pro-inflammatory role as activators of the cGAS-STING pathway. We recently proved them also as radiation-induced senescence messages for the abscopal effect mediation, demonstrating the need for a functional p53 for their production and release in A549 and H1299 tumour cells. However, little is known about their role under different stress conditions, especially in cancer cells.

**Methods:**

In this work, we open the investigation making use of automated quantitative imaging to characterize the hybrid subcellular distribution in HeLa cells grown under different oxygen pressures or exposed to different ionizing radiation doses. After cell imaging by confocal fluorescent microscopy, we apply automated imaging methods developed on purpose to quantify hybrid foci and nuclear cluster intensity, regional and local density and dimension.

**Results:**

We show that alteration of culture oxygenation increases hybrid cytoplasmic presence, especially when caused by an hyperoxic environment, with evident hybrid gathering at the cell membrane. Ionizing radiations always fail to increase hybrids, in accordance with the absence of functional p53 in HeLa cells. However, dose-dependent effects are still evident and suggest a threshold dose of 7.5 Gy for remarkable hybrid reduction.

**Conclusion:**

Together with our previous results, these data demonstrate for the first time that different types of stress can increase hybrid production in cancer cells and by at least two different pathways, one p53-dependent triggerable by ionizing radiations and one p53-independent triggerable by oxidative stress. Together, our findings provide a starting point for understanding hybrid role in tumour stress response.

## Introduction

RNA:DNA hybrids are secondary transcriptional products whose unwanted persistence or mislocation inside cell nucleus can lead to genomic instability and ultimately to many disease onset, including cancer [1, 2]. However, hybrid presence has also been confirmed inside the mitochondria [3, 4], extracellular vesicles (EVs) [5] and cytosol [6, 7]. While mitochondrial hybrids share many behaviours with their nuclear counterpart [3, 4, 8], cytosolic hybrids have an established pro-inflammatory role as activators of the cGAS-STING pathway that triggers the expression of inflammatory genes that can lead to cellular senescence or to the activation of defence mechanisms through the exportation of molecular messages in the tumour microenvironment [5, 8]. We have recently identified hybrids also as cargoes for EVs released by 10 Gy-irradiated A549 TP53+ cells, conveying a senescence message able to reach distant sites and mediate abscopal effect [5]. In general, it has been proposed that a variety of genetic and/or pharmacological perturbations can lead to the formation of the so-called harmful RNA:DNA hybrids, whose presence is also linked to different types of DNA damaging stresses [9–11].

Despite their increasing relevance, the rules that govern RNA:DNA hybrid formation and dynamics are still controversial, and technical improvements that allow the measurement of their turnover are required to distinguish and quantify stable and transient hybrids. The S9.6 antibody is one of the main solutions for hybrid detection and currently represents the only available antibody for hybrid detection by fluorescence microscopy. Though concerns about S9.6 specificity have been more than once raised [12, 13], when coupled with rigorous quantitative image analysis, it can still provide insights about the radiomorphometry of hybrid distribution, opening to phenomization of cell response to treatment. In this perspective, we have recently developed a novel tool for quantifying and mapping the local density of marked particles in bi-dimensional (2-D) images, including RNA:DNA hybrids immunostained with S9.6 antibody [14].

In this work, we expand our previous research [5] by firstly exploring how different stress conditions affect the subcellular distribution of RNA:DNA hybrids, hypothesizing the stress nature as a primary director of hybrid dynamics inside the cell. In particular, we use automated quantitative imaging to characterize the nuclear and cytoplasmic distribution of hybrids in HeLa after cell irradiation (IR) at different doses or after cell culturing at different oxygenation levels. To the best of our knowledge, this is the first work that finely investigates the subcellular distribution of RNA:DNA hybrids, not only in terms of radiometry, but also considering their aggregation and position inside the cell. Indeed, RNA:DNA hybrids are often quantified by fluorescence microscopy, but this usually involves only assessing their presence or basic intensity measurements [9, 15, 16]. With our approach, we show that hybrid presence is not increased by IR in p53-defective HeLa cells, thus confirming the phenomenon dependency on a functional p53. However, a threshold dose of 7.5 Gy for significant hybrid reduction is identifiable. Oppositely, hybrid presence is increased under oxidative conditions, and their aggregation (hereon, condensation) state is here suggested to depend on the oxygenation level. Although further experiments will be needed to confirm our preliminary findings, our data indicate for the first time the existence of at least one p53-independent pathway for stress-induced hybrid accumulation.

## Material and Methods

### Assessment of Results

This study investigates how different stress conditions affect the subcellular distribution of RNA:DNA hybrids in HeLa cells through automated quantitative imaging. At the regional level (i.e., whole nucleus and whole cytoplasm), hybrid abundance is quantified by features summarizing the hybrid number, dimension and brightness, whereas at the subregional (i.e., finely local) level, the hybrid absolute and relative (to each other) position is investigated by *local density analysis* [14]. Importantly, hybrid abundance must *not* here be intended as a measure of hybrid molecule number. In fact, it is not yet clear whether RNA:DNA hybrids may contain a specific consensus and if S9.6 possesses any intrinsic sequence specificity [17]. Moreover, S9.6 can bind to hybrids as short as 8 bp [18], while hybrid length has been reported to vary even beyond 50 bp [19]. This means that no assumption on the S9.6-hybrid binding stoichiometry can be made and that, therefore, it is impossible to count hybrid molecules with S9.6. Therefore, our analysis does not address the single molecule but a group of them, representing our analysis unit. To set the physical boundaries of such unit, we apply a geodesic principle to signal intensity to separates the overlapping “blobs” of hybrids into discrete *foci* of diverse dimension. With this setup, an increase in foci intensity still indicates an increase in S9.6-hybrid binding and therefore in hybrid abundance, intended as molecule number, length or both. To better describe hybrid abundance, besides foci intensity, we further measure regional hybrid foci number, while regional foci area and subregional local density are measured as indicators of hybrid position and condensation.

### Cell Culture and Treatment

Human cervix adenocarcinoma HeLa cells (ATCC, Manassas, VA, USA) are cultured in EMEM (ATCC) supplemented with 10% FBS (Euroclone, Milan, Italy), 1% penicillin/streptomycin (GE Healthcare, Milan, Italy) and 2% amphotericin B (Euroclone, Milano, Italy). Two groups of treatments are considered: the OX group, involving the alteration of culture oxygenation, and the IR group, involving the γ-irradiation of cells. The OX group comprises three different cycles of oxidative stress conditions: (a) 72 h of hypoxic culturing conditions (37°C, 1% O_2_) (HYPOX); (b) full cell growth under normoxic conditions (37°C, 21% O_2_), followed by 1 h of cell exposure to hyperbaric oxygen (37°C, 1.9 ATA HBO) and then 24 h of normoxic culturing conditions (HBO); and (c) a sequential combination of hypoxic culturing conditions (48 hours, 37°C, 1% O_2_) and cell exposure to hyperbaric oxygen (1 h, 37°C, 1.9 ATA HBO) (HYPOX+HBO). HBO treatment was carried out by a hyperbaric chamber expressly designed for preclinical studies, as previously described [20]. In particular, the air inside the chamber is replaced with 100% O_2_, and the pressure increased for 15 min until 1.9 ATA are reached and maintained for 1 h. Finally, decompression from 1.9 ATA back to atmospheric pressure is gradually performed over 15 min. The IR group comprises four different treatments, namely, the 1× cell irradiation at 2, 7.5, 10 or 20 Gy, as these are the IR doses routinely employed in fractionation schemes for the treatment of different tumour histotypes, including cervical cancer [21, 22]. Cells were irradiated using the linear accelerator Elekta Synergy Platform system (Elekta Oncology Systems, Stockholm, Sweden), as previously described [23]. Unirradiated cells, grown under normoxic conditions, are used as untreated control (UC) for both treatment groups.

### Fluorescence Microscopy

Cells are fixed and permeabilized with ice-cold methanol for 10 min and acetone for 1 min on ice, blocked with 2% BSA, stained with 1 µg/ml 4′,6-diamidino-2-phenylindole (DAPI) and incubated overnight at 4°C with primary anti-S9.6 antibody (1:100 dilution, Kerafast, Boston, MA, USA). After washing 5 times with PBS 1×, slides are incubated 1 h at room temperature with secondary goat anti-mouse Alexa Fluor 568 (1:250, Life Technologies, Carlsbad, CA, USA). Cells are imaged with an inverted confocal laser-scanning microscope Eclipse Ti2-e (Nikon Corporation, Tokyo, Japan) equipped with NIS-Elements software with laser power (LP) and spectra details as follows: DAPI (LP = 3.8 mW, λ_EX_ = 350 nm, λ_EM_ = 470 nm, detection window (DW) = 417-508 nm), Alexa Fluor 568 (LP = 0.92 mW, λ_EX_ = 578 nm, λ_EM_ = 603 nm, DW = 570-695 nm). Twelve-bit images are acquired with a Plan Apo 60×/1.4 oil objective (pinhole size = 28.1 µm, exposure time = 4.2 s) and a built-in Nikon A1 plus camera (5 fps with frame accumulation) with XYZ pixel size of 0.1 µm^3^ and an optical section number sufficient for the full scan of cell volumes. Each experiment has been performed once, with replicates number (i.e., imaged cells) as follows: 197 (UC), 200 (HBO), 314 (HYPOX), 169 (HYPOX-HBO), 35 (2 Gy), 79 (7.5 Gy), 32 (10 Gy) and 31 (20 Gy).

### Image Analysis

The hybrid foci analysis procedure is developed on purpose and implemented in MATLAB^®^ (R2019b v.9.7.0, The MathWorks, Natick, MA, USA) and ImageJ (NIH, Bethesda, MD, USA) and consists of the following steps: image segmentation, global features extraction and quantification, local density analysis and statistical analysis.

### Image Segmentation

After maximum intensity projection (MIP) image construction on top of the acquired sections, a semi-automated procedure is developed to separately address RNA:DNA hybrids in each imaged cytoplasm and nucleus. First, the nuclear region is adaptively and conservatively segmented by mean of DAPI staining, involving the computation of local median maps [24] in case of irradiated cells and local contrast enhancement [25] otherwise. Then, after segmenting the channel fusion image by thresholding at histogram mode, single cells are separated with watershed transform [26]. The use of logical operators permits to isolate single-cell nuclei and cytoplasms. Hybrid foci are finally segmented in each cell compartment by computing the local median map and thresholding it at the third quartile, applying an intermediate top-hat transform [27] in case of cytoplasm segmentation. Finally, single foci are geodesically reconstructed by dilation [28]. Additionally, hybrid nuclear clusters are separated from nuclear foci on a dimension criterion set after image pre-scouting and further distinguished in perinuclear and nucleolar clusters basing on their contiguity with the nuclear membrane.

### Global Feature Extraction and Quantification

Regional RNA:DNA hybrid distribution is globally described through quantification of regional foci and nuclear cluster intensity, number (normalized by the region area) and foci granulation index (FGI) (i.e., the percentage of regional foci having 1-pixel area). The FGI feature is specifically introduced in this study to quantify those hybrid foci occurring at a size close to the resolution burden, and it is hence not applicable, by definition, to nuclear clusters analysis.

### Local Density Analysis

Local density analysis is performed by computing the density distribution map (DDM) of each cell, as described in [14], using a minimal 3 × 3 search window*.* Each pixel can thus be assigned with a local density index (LDI) ranging from 0 for isolated pixels to 8 for full-connected ones, and distribution can be visualized and quantified in colormaps that associate each local density with a different colour.

### Statistical Analysis

Statistical analysis is performed in MATLAB^®^. Data deviation from normality is early verified by histogram inspection, followed by the Shapiro–Wilk test, based on which the discriminatory power of descriptors is assessed by either two-tail Student’s *t* test or Wilcoxon rank-sum test with Bonferroni correction for unequal sample size. *p* values < 0.05 are considered for statistical significance.

## Results and Discussion

### Stress Conditions Homogenize Hybrid Distributions

With few exceptions, all tested conditions homogenize the cell response to treatment, by lowering the variance associated with considered features.

As reported in Table 1, all treatments regularize the foci number by reducing its associated variance of about one order of magnitude with respect to the UC. All treatments, except for the 10 Gy treatment, regularize the FGI. All treatments, except for the 2 Gy and the HBO treatment, regularize the average foci intensity. For both the FGI and the foci intensity, the variance increases with the measurement magnitude. Finally, the variance of perinuclear and nucleolar clusters number and intensity is stable with treatments or proportional to the measure magnitude (data not shown). Arguably, this last observation could be biased by the greater object size that reduces the number of clusters that can fit into a single nucleus and can hence be separately analysed.

**Table 1:** Global foci feature variance under tested conditions

		Foci number		FGI		Foci intensity	
	*n*	Nucleus	Cytoplasm	Nucleus	Cytoplasm	Nucleus	Cytoplasm
UC	197	1.27 × 10^-5^	1.63 × 10^-5^	4.98 × 10^-3^	2.45× 10^-3^	1.43 × 10^5^	2.72 × 10^5^
HYPOX	314	5.61 × 10^-6^	4.27 × 10^-6^	2.76 × 10^-3^	1.74 × 10^-3^	8.13 × 10^4^	9.84 × 10^4^
HYPOX+HBO	169	5.65 × 10^-6^	3.08 × 10^-6^	3.76 × 10^-3^	2.47 × 10^-3^	1.30 × 10^5^	1.96 × 10^5^
HBO	200	7.39 × 10^-6^	5.33 × 10^-6^	2.66 × 10^-3^	1.42 × 10^-3^	3.12 × 10^5^	3.14 × 10^5^
2 Gy	135	1.74 × 10^-6^	1.03 × 10^-6^	7.33 × 10^-4^	7.61 × 10^-4^	1.83 × 10^5^	3.78 × 10^5^
7.5 Gy	79	1.66 × 10^-6^	1.54 × 10^-6^	9.03 × 10^-4^	2.08 × 10^-4^	3.22 × 10^4^	7.01 × 10^4^
10 Gy	32	1.97 × 10^-6^	5.65 × 10^-6^	6.39 × 10^-3^	4.81 × 10^-3^	3.10 × 10^4^	2.07 × 10^4^
20 Gy	31	3.79 × 10^-6^	2.30 × 10^-6^	5.30 × 10^-4^	1.63 × 10^-4^	5.12 × 10^4^	1.90 × 10^4^

*n*, sample size. *FGI*, foci granulation index. *UC*, untreated control; *HYPOX*, hypoxic treatment; *HYPOX+HBO*, sequential hypoxic and hyperbaric oxygen treatment

### Perturbation of Culture Oxygenation Increases RNA:DNA Hybrid Production and Condensation in p53-Defective Cells

**Fig. 1:**
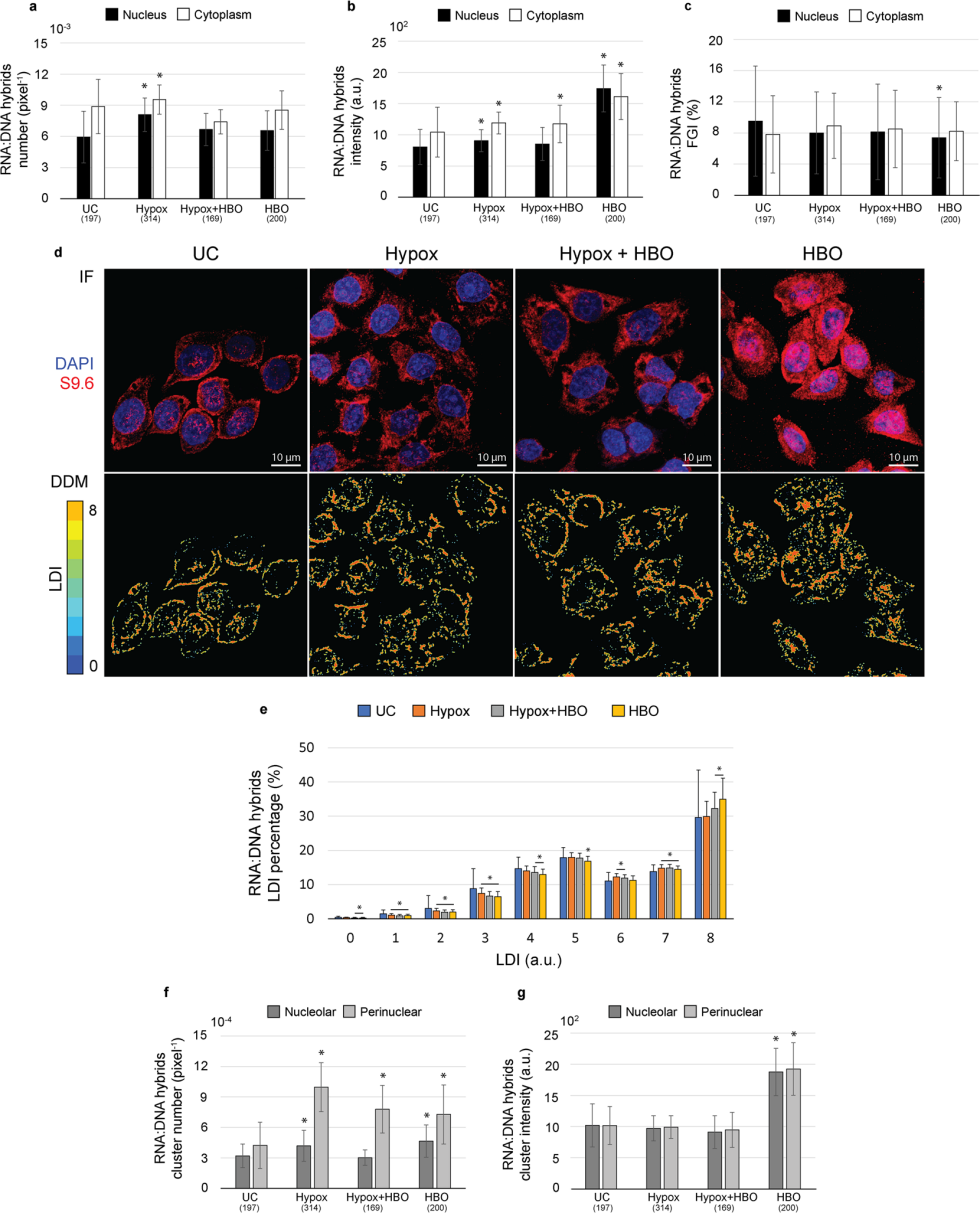
Effects of OX treatments on RNA:DNA hybrid subcellular distribution in HeLa cells. Bar graphs of hybrid foci density (**a**), intensity (**b**) and FGI (**c**) in the main cell compartments, reported as median ± MAD (**a**, **b**) and as mean ± STD (**c**). (**d)** Representative RGB MIPs (IF, top) and DDMs (bottom) of HeLa cells cultured under normoxic (UC), hypoxic (HYPOX), hyperoxic (HBO) or combined (HYPOX+HBO) conditions, stained for DNA (blue) and immunolabeled against RNA:DNA hybrids (red). (**e)** Bar graph of LDI percentages, reported as mean ± STD. Bar graphs of hybrid perinuclear and nucleolar cluster density (**f**) and intensity (**g**) in the main cell compartments, reported as median ± MAD. * *p*<0.05 for statistical comparison with UC by Wilcoxon rank-sum test (**a**, **b**, **f**, **g**) or two-tail Student’s *t* test (**c**, **e**)

Perturbation of oxygenic culture conditions globally increases the RNA:DNA hybrid foci number (Fig. 1a) and intensity (Fig. 1b), in both nucleus and cytoplasm.

Specifically, hybrid foci number (i.e., density) is significantly increased under hypoxic conditions, especially inside cell nucleus (Fig. 1a) (+36%, *p*=10^−13^), whereas hybrid intensity is increased after every perturbation of culture oxygenation, especially after HBO exposure and inside cell nucleus, where it more than doubles (Fig. 1b) (+117%, *p*=10^−42^). As cytoplasm hybrids are considered to derive from nuclear displacement [29], increase in foci number and intensity value in both compartments likely reflects an increase in hybrid nuclear production and consequent exportation to cytoplasm, with no remarkable constraints [6]. The fact that hypoxic and hyperoxic conditions promote such similar response could be ascribed to their common capability to promote intracellular oxidative stress through massive production of reactive oxygen species (ROS) [20, 30]. Different culture oxygenation levels likely lead to a different intracellular concentration of ROS that directly determine the extent of DNA breaks, from which hybrids are known to arise [31, 32]. Moreover, ROS and hybrid involvement in a common pathway is supported by the pro-inflammatory role of both [5, 20, 33] and by data collected for both the mitochondrial [34, 35] and the nuclear genome [9], which report an increase in RNA:DNA hybrids and R-loops after oxidative stress. However, as hyperoxic and hypoxic responses always significantly differ from each other, the hybrid production can be hypothesized to be affected or even regulated by culture oxygen concentration. In line with this, the cell culturing under sequential hypoxic and hyperoxic conditions showed to almost never alter hybrid foci number or intensity, thus indicating that hyperoxic conditions can alleviate the stress caused on cells by hypoxia, as well acknowledged in HBO therapy [36]. Accordingly, HBO was also recently proposed for the treatment of recurrent glioblastoma in association with radiotherapy in absence of druggable driver mutations [37].

In both cell nucleus and cytoplasm, under hypoxic conditions, hybrid foci are more (Fig. 1a) (+18% on average, *p*<10^−8^) but much less bright (Fig. 1b) (−92% inside nucleus, *p*=10^−47^, −36% inside cytoplasm, *p*=10^−22^) than under hyperoxia. This discrepancy points at different foci condensation states for the two conditions that could depend on the oxygen level. To investigate the cause of such difference, we introduce the foci granulation index (FGI) that is a measure of the percentage of 1-pixel foci. Low and not significant differences between cytoplasmic hybrid FGI under hypoxic and hyperoxic conditions (Fig. 1c) (8% average difference, *p*≥0.06) indicate that such imbalance between foci number and intensity is not due to a poor image resolution, insufficient to capture hybrids smaller than 1 pixel, but derives from a different foci distribution. To deepen the investigation, we perform a local density analysis by creating density distribution maps (DDMs) [14]. When coupled with global measurements, DDMs permit to investigate whether regional phenomena are mirrored at the subregional local level or if a subregional homogeneity is wrongly assumed and summarized in a regional measure. Accordingly, DDMs revealed a different distribution of local densities under hyperoxic and hypoxic conditions (Fig. 1d). With respect to hypoxia, hyperoxia is significantly enriched in maximum local density presence (Fig. 1e) (LDI=8, +17%, *p*=10^−24^) at the expense of the lowest ones (Fig. 1e) (LDI=0–7, −11% on average, *p*<10^−4^), indicating more densely packed hybrids under hypoxia. A similar but milder response than hyperoxia are reported for the combinatory treatment. Qualitatively, DDMs depict more similarities between signal distributions under hypoxic and hyperoxic conditions. In particular, the hybrid gathering at the cell membrane in UC cells is coupled to a scattered hybrid signal in cell cytoplasm after every treatment of the OX group, especially the hyperoxic one. Moreover, DDMs highlight dense hybrid clusters inside hyperoxic nuclei, as foreseen by FGI decrease. Accordingly, the analysis of nuclear clusters reveals an increase in perinuclear cluster number after every perturbation of culture oxygenation (Fig. 1f) (+97% on average, *p*<10^−13^) which also increase in intensity under hyperoxic conditions (Fig. 1g) (+89%, *p*=10^−33^). Similarly, hyperoxia also increases the hybrid nucleolar cluster intensity (Fig. 1g) (+84%, *p*=10^−22^) and number (Fig. 1f) (+45%, *p*=10^−5^), this latter variation also being induced by hypoxia (Fig. 1f) (+31%, *p*=10^−5^). Overall, oxidative stress seems to promote nuclear hybrid production and condensation in clusters, especially in hyperoxic culture conditions. Nucleolar clusters can be reasonably hypothesized to identify with cell nucleoli, in which hybrids have been often reported to accumulate, especially in stressed cancer cells [5, 38, 39]. On the other hand, an enrichment in perinuclear clusters may hint at over-produced hybrids that accumulate at the nuclear membrane, before being exported to cytoplasm [6].

### Cell Irradiation Decreases RNA:DNA Hybrid Production and Affects Their Condensation in a Dose-Dependent Fashion in p53-Defective Cells

**Fig. 2:**
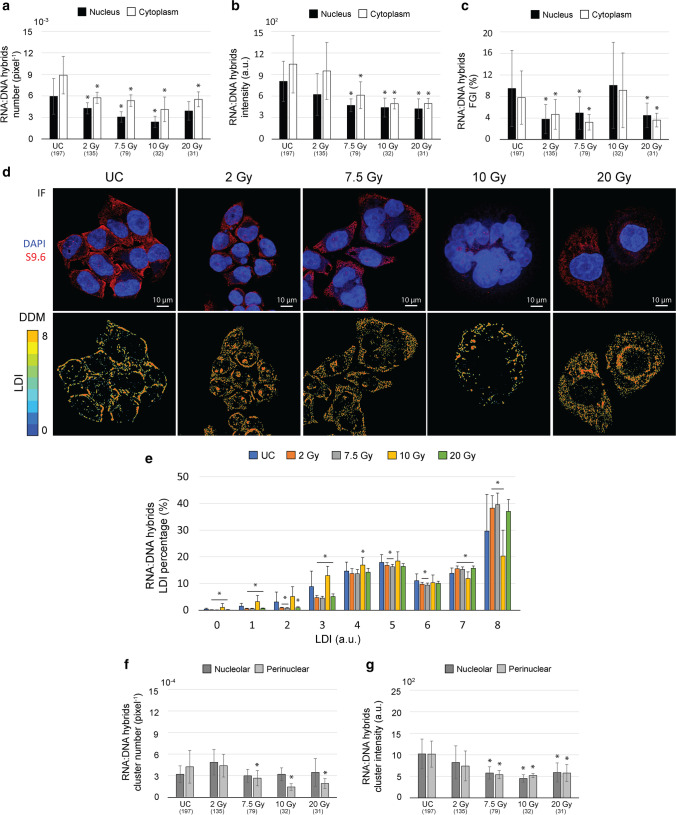
Effects of IR treatments on RNA:DNA hybrid subcellular distribution in HeLa cells. Bar graphs of hybrid foci density (**a**), intensity (**b**) and FGI (**c**) in the main cell compartments, reported as median ± MAD (**a**, **b**) and as mean ± STD (**c**). (**d)** Representative RGB MIPs (IF, up) and DDMs (down) of HeLa cells unirradiated (UC) or 1× irradiated at 2 Gy, 7.5 Gy, 10 Gy or 20 Gy, stained for DNA (blue) and immunolabeled against RNA:DNA hybrids (red). (**e)** Bar graph of LDI percentages, reported as mean ± STD. Bar graphs of hybrid perinuclear and nucleolar cluster density (**f**) and intensity (**g**) in the main cell compartments, reported as median ± MAD. * *p*<0.05 for statistical comparison with UC by Wilcoxon rank-sum test (**a**, **b**, **f**, **g**) or two-tail Student’s *t* test (**c**, **e**)

Cell irradiation globally decreases the RNA:DNA hybrid density (Fig. 2a) and intensity (Fig. 2b) in HeLa cells, again showing similar trends for cell nucleus and cytoplasm.

Specifically, while a dose of 2 Gy decreases hybrid foci number (i.e., density) (Fig. 2a) (−32% on average, *p*<10^−5^), higher doses have both hybrid number (Fig. 2a) (−48% on average, *p*<10^−5^) and intensity (Fig. 2b) (−47% on average, *p*<10^−7^). As for OX group treatments, the simultaneous decrease in hybrid foci number and intensity in both nucleus and cytoplasm suggests for an IR-induced decline in their production and consequent exportation to cytoplasm, either actively arising from a lowering of their production or passively arising from its simple hampering. Also, the exception represented by the 2 Gy dose suggests it as a too mild stress for hybrid significant alteration, appreciable only from 7.5 Gy on.

To investigate foci condensation in irradiated cells, we analyse FGI and DDMs. Consistently with their regional number, hybrid FGI is significantly decreased after irradiation at all tested doses in both cell nucleus and cytoplasm (Fig. 2c) (−52% on average, *p*<10^−4^), except at 10 Gy. Accordingly, only 10 Gy-irradiated cells display a local hybrid distribution comparable to that of UC (Fig. 2d) (DDMs), with dense accumulation of cytoplasmic hybrids at the nuclear and cell membrane. Conversely, at 2 Gy, 7.5 Gy and 20 Gy, hybrids appear more and more scattered in the cell cytoplasm, with nuclear clusters appearing at 2 Gy and then gradually disappearing with dose from nuclei. Quantitatively, doses of 2 Gy, 7.5 Gy and 20 Gy induce a strong increase of high local densities (Fig. 2e) (LDI=7–8, +21% on average, *p*<10^−6^), at the expense of lower ones (Fig. 2e) (LDI=1–6, −41% on average, *p*<0.002). Conversely, a dose of 10 Gy increases the presence of low and medium densities (Fig. 2e) (LDI=0–5, +69% on average, *p*<10^−3^) while decreasing the higher ones (Fig. 2e) (LDI=6–8, −17% on average, *p*<10^−3^). Therefore, the DDMs and the FGI indicate that cell irradiation induces hybrid condensation, except for the 10 Gy dose. The fact that this is not mirrored by regional number measurements (Fig. 2a) reveals the heterogeneity of cell response that produces subregional variations in foci number and intensity that cannot be summarized in a regional measure, at least after 10 Gy irradiation. Finally, an IR dose of at least 7.5 Gy decreases perinuclear cluster number (Fig. 2f) (−56% on dose average, *p*<0.002) and both perinuclear and nucleolar clusters intensity (Fig. 2g) (−47% on average, *p*<10^−3^). Again, the 2 Gy dose is suggested to be a too mild stress for hybrid remarkable alteration, while higher doses seem to lead to the complete removal of hybrids from the nucleus.

All considered, these data confirm the requirement for a functional p53 for boosting hybrid production after γ-IR in tumour cells. Indeed, while cell IR increased hybrid production in A549 TP53+ cells [5], it oppositely decreases hybrid presence in HeLa cells, where p53 is inactivated by the HPV E6 protein [40]. Furthermore, ionizing radiation can be hypothesized to reduce RNA:DNA hybrid production in a dose-dependent manner, at least in HeLa cells. Dose dependency of hybrid production has been only marginally investigated before [5, 41]. In particular, our previous work identified an IR dose of 10 Gy as the minimum dose capable to significantly increase hybrid production and exportation from cells, through EVs, in A549 TP53+ cells [5]. Our data indicate that in HeLa cells, this threshold may be lowered to 7.5 Gy that could act as a less powerful yet sufficient stimulus for hybrid boosting, as already reported for dermal fibroblast cultures [29]. Accordingly, an IR dose of 2 Gy slightly perturbs hybrid distribution by only inducing local condensation. However, if we assume a linear relation between the delivered IR dose and hybrid production, an exception to such linearity is represented by the 10 Gy dose, which causes hybrid number and intensity to reach their minimum while accumulating under the cell membrane. According to our previous findings [5], this pattern suggests that, despite p53 absence, a dose of 10 Gy may be sufficient to promote hybrid release from cells. In this scenario, hybrid gathering at the cell membrane could be read as a pro-exportation event that mirrors EV accumulation in multivesicular bodies (MVBs), prior to exit the cell [42]. Though compelling and logically solid, these considerations need to be thoroughly verified by further experiments, such as EV content analysis, also considering further tumour cell types.

Finally, also the expression of the DNA exonuclease TREX1 has recently been proved to be dose dependently induced by ionizing radiations [41]. Given TREX1 capability to remove cytosolic endogenous DNA and hybrids [43], dose dependency of RNA:DNA hybrid accumulation should be also examined in the perspective of being just a mirror of their degradation.

## Conclusions

The relevance of RNA:DNA hybrids is still under debate, as well their involvement in genomic instability. Current models propose that a variety of genetic and/or pharmacological perturbations can lead to RNA:DNA hybrid formation [38], and we have recently shown that this is the case of A549 TP53+ tumour cells subjected to 10 Gy and 20 Gy IR [5]. These doses have been reported to induce an immune response through the innate and adaptive immune system, and they are currently receiving considerable attention from the scientific community [44, 45]. In this work, we have extended the investigation to different stress conditions and one more cell line. Our results show that oxygen-based and irradiation-based treatments induce opposite responses in HeLa cells that bear an inactivated p53. On one hand, oxidative stress increases RNA:DNA hybrids, especially if caused by a hyperoxic environment, while on the other, the hybrid presence decreases as the irradiation dose rises. Implications of our findings are manyfold. First, being differently affected by different types of stresses, hybrid presence can be hypothesized to be stress-related but not stress-specific. Secondly, our data confirm that ionizing radiation capability to boost hybrid production positively depends on the presence of a functional p53. This implies the existence of at least a p53-independent pathway for hybrid production stimulation that can be elicited by oxidative stress stimuli. In particular, our results suggest that RNA:DNA hybrids could be strongly induced in the absence of a functional p53 by hyperbaric oxygen, a well-established treatment used as an adjunctive therapy in many disease settings. Furthermore, our results indicate that different types of stress can promote hybrid production, consistently with these molecule capability to activate the pro-inflammatory cGAS-STING pathway [2]. Nevertheless, although our experiments referred to three tumour cell lines only, the analyses hint at a cell line-specific sensitivity to ionizing radiations that impacts on hybrid distribution. Finally, our DDM quantification of hybrid accumulation at the main cell membranes preliminarily suggest RNA:DNA hybrid exportation from cell nucleus to cytoplasm [29] and then to the microenvironment, where they have been found to convey a message of senescence capable of triggering innate immune response [5]. Much remains to be investigated of hybrid roles and functions, and these results would surely benefit from a molecular confirmation, possibly in diverse tumour cell types. Nonetheless, this work suggests hybrid subcellular distribution and the automated quantitative imaging approach as a starting point for a comprehensive phenomization of cell response to stress.
